# Crystal structure of 2-hy­droxy­imino-2-(pyridin-2-yl)-*N*′-[1-(pyridin-2-yl)ethyl­idene]acetohydrazide

**DOI:** 10.1107/S1600536814025793

**Published:** 2014-11-29

**Authors:** Maxym O. Plutenko, Rostislav D. Lampeka, Matti Haukka, Ebbe Nordlander

**Affiliations:** a Department of Chemistry, National Taras Shevchenko University, Volodymyrska Street 64, 01601 Kyiv, Ukraine; b Department of Chemistry, University of Jyvaskyla, PO Box 35, FI-40014 Jyvaskyla, Finland; c Inorganic Chemistry, Center for Chemistry and Chemical Engineering, Lund University, Box 124, SE-221 00 Lund, Sweden

**Keywords:** crystal structure, hy­droxy­imino, acetohydrazide, pyridyl­ethyl­idene, hydrogen bonding, π–π stacking inter­actions

## Abstract

The mol­ecule of the title compound, C_14_H_13_N_5_O_2_, is approximately planar (r.m.s deviation for all non-H atoms = 0.093 Å), with the planes of the two pyridine rings inclined to one another by 5.51 (7)°. The oxime group is *syn* to the amide group, probably due to the formation of an intra­molecular N—H⋯N hydrogen bond that forms an *S*(6) ring motif. In the crystal, mol­ecules are linked by pairs of bifurcated O—H⋯(O,N) hydrogen bonds, forming inversion dimers. The latter are linked *via* C—H⋯O and C—H⋯N hydrogen bonds, forming sheets lying parallel to (502). The sheets are linked *via* π–π stacking inter­actions [inter-centroid distance = 3.7588 (9) Å], involving the pyridine rings of inversion-related mol­ecules, forming a three-dimensional structure.

## Abstract 2

The mol­ecule of the title compound is approximately planar with the planes of the two pyridine rings inclined to one another by 5.51 (7)°. In the crystal, mol­ecules are linked by bifurcated O—H⋯(O,N) hydrogen bonds, forming inversion dimers, which are in turn linked *via* C—H⋯O and C—H⋯N hydrogen bonds, forming sheets lying parallel to (502).

## Chemical context   

Polynuclear oxime-containing ligands have attracted considerable inter­est because of their ability to act as efficient bridging ligands and for their tendency to form polynuclear metal complexes (Penkova *et al.*, 2010 ▶; Pavlishchuk *et al.*, 2010 ▶, 2011 ▶). The presence of additional non-oxime donor functions (*e.g.* hydrazide, azomethine, pyridine) in the ligand mol­ecule favours the formation of metal complexes with strong magnetic exchange inter­actions between the metal ions (Pavlishchuk *et al.*, 2011 ▶), and complexes which efficiently stabilize unusual high oxidation states of 3*d* metal ions (Kanderal *et al.*, 2005 ▶; Fritsky *et al.*, 1998 ▶, 2006 ▶). As a part of our research study, we present the structure of the title compound, which contains several donor functions of a different nature; oxime, hydrazide, and two different pyridine groups.
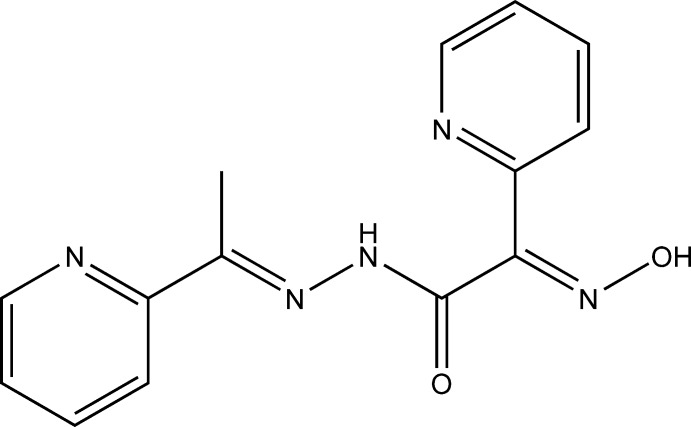


## Structural commentary   

**Figure 1:**
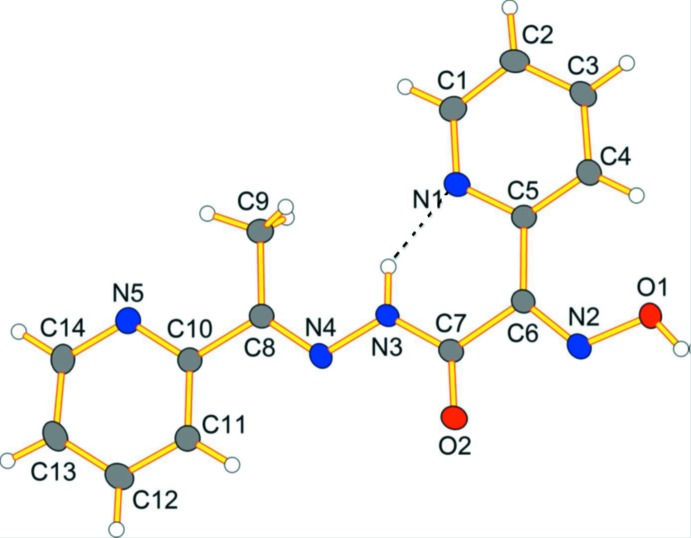
A view of the mol­ecular structure of the title compound, showing the atom labelling. Displacement ellipsoids are drawn at the 50% probability level. The intra­molecular N—H⋯N hydrogen bond is shown as a dashed line (see Table 1 ▶ for details).

The mol­ecular structure of the title compound is illustrated in Fig. 1 ▶. The mol­ecule is approximately planar (r.m.s deviation for all non-H atoms = 0.093 Å) with the maximum deviations from the mean plane being 0.255 (1) Å for atom N1, and 0.198 (1) Å for atom O1. The two pyridine rings (N1/C1–C5) and N5/C10–C14) are inclined to one another by 5.51 (7)°. The N2—O1 [1.3691 (14) Å] and C6—N2 [1.2866 (17) Å] bond lengths of the oxime group have typical values (Fritsky *et al.*, 1998 ▶). The pyridine N atom, N1, is situated in an *anti* position with respect to the azomethine group, in accordance with the structures of earlier synthesized ligands of this type (Plutenko *et al.*, 2011 ▶, 2013 ▶).

The N4—N3, N3—C7 and C7—O2 bond lengths of the hydrazide group are 1.3776 (16), 1.3471 (18) and 1.2269 (17) Å, respectively, typical for protonated moieties of this type (Plutenko *et al.*, 2011 ▶, 2013 ▶). The oxime group is situated in a *syn* position with respect to the amide group, in contrast to earlier synthesized ligands of this type (Plutenko *et al.*, 2012 ▶, 2013 ▶). Such a disposition of these moieties is atypical for amide derivatives of 2-hy­droxy­imino­propanoic acid (Onindo *et al.*, 1995 ▶; Sliva *et al.*, 1997 ▶; Duda *et al.*, 1997 ▶). It can be explained by the presence of an intra­molecular N3—H3⋯N1 hydrogen bond in which the azomethine N atom, N3, acts as donor and the pyridine N atom, N1, acts as an acceptor (Fig. 1 ▶ and Table 1 ▶).

## Supra­molecular features   

**Figure 2:**
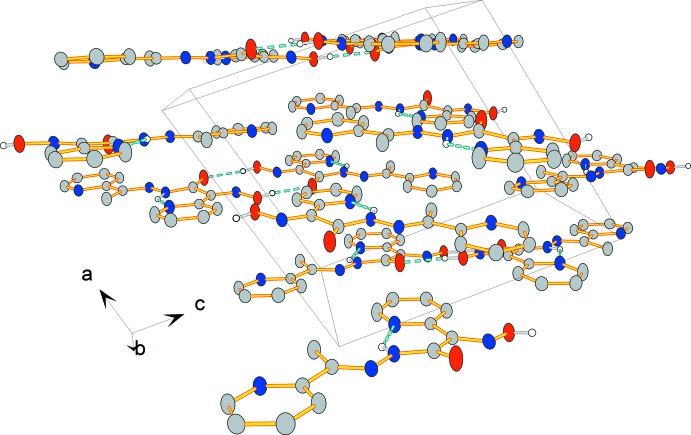
Crystal packing of the title compound viewed along the *b* axis. Hydrogen bonds are indicated by dashed lines (see Table 1 ▶ for details). H atoms not involved in hydrogen bonds have been omitted for clarity.

In the crystal, mol­ecules are linked by pairs of bifurcated O—H⋯(O,N) hydrogen bonds forming inversion dimers (Fig. 2 ▶ and Table 1 ▶). The dimers are linked *via* C—H⋯O and C—H⋯N hydrogen bonds, forming sheets lying parallel to plane (502). The sheets are linked *via* π–π stacking inter­actions, forming a three-dimensional structure [*Cg*1⋯*Cg*2^i^ = 3.7588 (9) Å; *Cg*1 and *Cg*2 are the centroids of pyridine rings N1/C1–C5 and N5/C10–C14, respectively; symmetry code: (i) −*x* + 1, −*y* + 2, −*z* + 1].

## Database survey   

The crystal structures of two very similar compounds have been reported, *viz.* 2-hy­droxy­imino-*N*′-(1-(pyridin-2-yl)ethyl­idene)propano­hydrazide (Moroz *et al.*, 2009 ▶) and two polymorphs of 2-(3,5-dimethyl-1*H*-pyrazol-1-yl)-2-(hy­droxyimino)-*N*′-(1-(pyridin-2-yl) ethyl­idene)acetohydrazide (Plutenko *et al.*, 2012 ▶, 2013 ▶).

## Synthesis and crystallization   

A solution of 2-hy­droxy­imino-2-(pyridin-2-yl)acetohydrazide (0.36 g, 2 mmol), prepared according to a published procedure (Zyl *et al.*, 1961 ▶; Kolar *et al.*, 1991 ▶), in methanol (20 ml) was treated with 2-acetyl­pyridine (0.242 g, 2 mmol) and the mixture was heated under reflux for 3 h. After cooling, the solvent was evaporated under vacuum and the resulting product was recrystallized from methanol, giving colourless block-like crystals of the title compound (yield 0.52 g; 92%).

## Refinement   

Crystal data, data collection and structure refinement details are summarized in Table 2 ▶. The N—H and O—H hydrogen atoms were located in difference Fourier maps and freely refined. The C-bound H atoms were positioned geometrically and constrained to ride on their parent atoms, with C—H = 0.95–0.98 Å, and with *U*_iso_ = 1.5*U*_eq_(C) for methyl H atoms and = 1.2*U*_eq_(C) for other H atoms.

## Supplementary Material

Crystal structure: contains datablock(s) I. DOI: 10.1107/S1600536814025793/su5024sup1.cif

Structure factors: contains datablock(s) I. DOI: 10.1107/S1600536814025793/su5024Isup2.hkl

Supporting information file. DOI: 10.1107/S1600536814025793/su5024Isup3.mol

Supporting information file. DOI: 10.1107/S1600536814025793/su5024Isup4.cml

CCDC reference: 1035993

Additional supporting information:  crystallographic information; 3D view; checkCIF report

## Figures and Tables

**Table 1:** Hydrogen-bond geometry (, )

*D*H*A*	*D*H	H*A*	*D* *A*	*D*H*A*
N3H3N1	0.89(2)	1.84(2)	2.6126(17)	144(2)
O1H1O2^i^	0.93(2)	1.98(2)	2.8327(14)	151(2)
O1H1N2^i^	0.93(2)	2.13(2)	2.8489(16)	133(2)
C2H2O2^ii^	0.95	2.54	3.1985(15)	127
C3H3*A*O1^iii^	0.95	2.56	3.4755(15)	163
C13H13N5^iv^	0.95	2.46	3.3811(19)	163

Symmetry codes: (i) 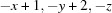; (ii) 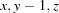; (iii) 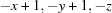; (iv) 
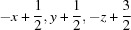
.

**Table 2:** Experimental details

Crystal data	
Chemical formula	C_14_H_13_N_5_O_2_
*M*_r_	283.29
Crystal system, space group	Monoclinic, *P*2_1_/*n*
Temperature (K)	123
*a*, *b*, *c* ()	11.4319(9), 9.3598(4), 12.4297(9)
()	105.016(3)
*V* (^3^)	1284.57(15)
*Z*	4
Radiation type	Mo *K*
(mm^1^)	0.10
Crystal size (mm)	0.27 0.15 0.14

Data collection	
Diffractometer	Bruker Kappa APEXII CCD
Absorption correction	Multi-scan (*SADABS*; Sheldrick, 1996 ▶)
*T*_min_, *T*_max_	0.973, 0.986
No. of measured, independent and observed [*I* > 2(*I*)] reflections	7900, 2850, 2226
*R*_int_	0.032
(sin /)_max_ (^1^)	0.651

Refinement	
*R*[*F* ^2^ > 2(*F* ^2^)], *wR*(*F* ^2^), *S*	0.044, 0.115, 1.05
No. of reflections	2850
No. of parameters	196
H-atom treatment	H atoms treated by a mixture of independent and constrained refinement
_max_, _min_ (e ^3^)	0.33, 0.22

Computer programs: *APEX2* and *SAINT* (Bruker, 2010 ▶), *SHELXS97* and *SHELXL97* (Sheldrick, 2008 ▶), *DIAMOND* (Brandenburg, 2008 ▶) and *PLATON* (Spek, 2009 ▶).

## supplementary crystallographic information

### Crystal data

**Table:**

C_14_H_13_N_5_O_2_	*F*(000) = 592
*M**_r_* = 283.29	*D*_x_ = 1.465 Mg m^−^^3^
Monoclinic, *P*2_1_/*n*	Mo *K*α radiation, λ = 0.71073 Å
Hall symbol: -P 2yn	Cell parameters from 12859 reflections
*a* = 11.4319 (9) Å	θ = 1.0–27.5°
*b* = 9.3598 (4) Å	µ = 0.10 mm^−^^1^
*c* = 12.4297 (9) Å	*T* = 123 K
β = 105.016 (3)°	Block, colourless
*V* = 1284.57 (15) Å^3^	0.27 × 0.15 × 0.14 mm
*Z* = 4	

### Data collection

**Table:**

Bruker Kappa APEXII CCD diffractometer	2850 independent reflections
Radiation source: fine-focus sealed tube	2226 reflections with *I* > 2σ(*I*)
Horizontally mounted graphite crystal monochromator	*R*_int_ = 0.032
Detector resolution: 16 pixels mm^-1^	θ_max_ = 27.6°, θ_min_ = 3.6°
φ scans and ω scans with κ offset	*h* = −14→7
Absorption correction: multi-scan (*SADABS*; Sheldrick, 1996)	*k* = −11→12
*T*_min_ = 0.973, *T*_max_ = 0.986	*l* = −13→16
7900 measured reflections	

### Refinement

**Table:**

Refinement on *F*^2^	Primary atom site location: structure-invariant direct methods
Least-squares matrix: full	Secondary atom site location: difference Fourier map
*R*[*F*^2^ > 2σ(*F*^2^)] = 0.044	Hydrogen site location: inferred from neighbouring sites
*wR*(*F*^2^) = 0.115	H atoms treated by a mixture of independent and constrained refinement
*S* = 1.05	*w* = 1/[σ^2^(*F*_o_^2^) + (0.0444*P*)^2^ + 0.5602*P*] where *P* = (*F*_o_^2^ + 2*F*_c_^2^)/3
2850 reflections	(Δ/σ)_max_ < 0.001
196 parameters	Δρ_max_ = 0.33 e Å^−^^3^
0 restraints	Δρ_min_ = −0.22 e Å^−^^3^

### Special details

**Table:**

Geometry. All e.s.d.'s (except the e.s.d. in the dihedral angle between two l.s. planes) are estimated using the full covariance matrix. The cell e.s.d.'s are taken into account individually in the estimation of e.s.d.'s in distances, angles and torsion angles; correlations between e.s.d.'s in cell parameters are only used when they are defined by crystal symmetry. An approximate (isotropic) treatment of cell e.s.d.'s is used for estimating e.s.d.'s involving l.s. planes.
Refinement. Refinement of *F*^2^ against ALL reflections. The weighted *R*-factor *wR* and goodness of fit *S* are based on *F*^2^, conventional *R*-factors *R* are based on *F*, with *F* set to zero for negative *F*^2^. The threshold expression of *F*^2^ > σ(*F*^2^) is used only for calculating *R*-factors(gt) *etc*. and is not relevant to the choice of reflections for refinement. *R*-factors based on *F*^2^ are statistically about twice as large as those based on *F*, and *R*- factors based on ALL data will be even larger.

### Fractional atomic coordinates and isotropic or equivalent isotropic displacement parameters (Å^2^)

**Table:**

	*x*	*y*	*z*	*U*_iso_*/*U*_eq_	
O1	0.53771 (10)	0.81661 (11)	0.01882 (9)	0.0275 (3)	
H1	0.547 (2)	0.873 (3)	−0.0406 (19)	0.061 (7)*	
O2	0.41325 (12)	1.10609 (11)	0.18548 (9)	0.0365 (3)	
N1	0.42020 (13)	0.68309 (13)	0.29508 (10)	0.0269 (3)	
N2	0.49655 (12)	0.91005 (12)	0.08547 (10)	0.0227 (3)	
N3	0.39671 (12)	0.95674 (13)	0.32531 (10)	0.0217 (3)	
H3	0.3983 (18)	0.865 (2)	0.3433 (16)	0.045 (6)*	
N4	0.36523 (11)	1.06522 (12)	0.38730 (9)	0.0211 (3)	
N5	0.27415 (12)	1.10442 (13)	0.63724 (10)	0.0249 (3)	
C1	0.40835 (17)	0.54907 (16)	0.32784 (14)	0.0332 (4)	
H1A	0.3841	0.5347	0.3946	0.040*	
C2	0.42966 (11)	0.43023 (11)	0.26956 (9)	0.0279 (3)	
H2	0.4195	0.3363	0.2946	0.033*	
C3	0.46609 (11)	0.45322 (11)	0.17415 (9)	0.0261 (3)	
H3A	0.4818	0.3742	0.1320	0.031*	
C4	0.48006 (15)	0.59117 (15)	0.13888 (12)	0.0258 (3)	
H4	0.5061	0.6074	0.0732	0.031*	
C5	0.45529 (13)	0.70606 (14)	0.20141 (11)	0.0196 (3)	
C6	0.46209 (13)	0.85875 (14)	0.16827 (11)	0.0198 (3)	
C7	0.42130 (14)	0.98666 (14)	0.22744 (12)	0.0225 (3)	
C8	0.33284 (13)	1.02516 (14)	0.47426 (11)	0.0206 (3)	
C9	0.32455 (16)	0.87266 (15)	0.50997 (13)	0.0282 (4)	
H9A	0.2782	0.8166	0.4466	0.042*	
H9B	0.2838	0.8694	0.5702	0.042*	
H9C	0.4062	0.8327	0.5363	0.042*	
C10	0.30190 (13)	1.14279 (14)	0.54298 (11)	0.0200 (3)	
C11	0.30120 (14)	1.28513 (15)	0.50943 (12)	0.0253 (3)	
H11	0.3217	1.3094	0.4424	0.030*	
C12	0.27019 (15)	1.39034 (16)	0.57523 (13)	0.0281 (3)	
H12	0.2690	1.4879	0.5539	0.034*	
C13	0.24098 (14)	1.35182 (16)	0.67237 (12)	0.0257 (3)	
H13	0.2188	1.4216	0.7190	0.031*	
C14	0.24502 (15)	1.20881 (16)	0.69948 (12)	0.0275 (3)	
H14	0.2259	1.1824	0.7667	0.033*	

### Atomic displacement parameters (Å^2^)

**Table:**

	*U*^11^	*U*^22^	*U*^33^	*U*^12^	*U*^13^	*U*^23^
O1	0.0449 (7)	0.0188 (5)	0.0253 (6)	0.0028 (4)	0.0206 (5)	−0.0006 (4)
O2	0.0691 (9)	0.0166 (5)	0.0338 (6)	0.0044 (5)	0.0315 (6)	0.0034 (4)
N1	0.0415 (8)	0.0172 (6)	0.0263 (6)	0.0000 (5)	0.0165 (6)	−0.0005 (5)
N2	0.0321 (7)	0.0180 (6)	0.0197 (6)	0.0003 (5)	0.0100 (5)	−0.0028 (4)
N3	0.0323 (7)	0.0147 (6)	0.0213 (6)	0.0013 (5)	0.0128 (5)	−0.0003 (4)
N4	0.0278 (7)	0.0173 (6)	0.0203 (6)	0.0010 (5)	0.0102 (5)	−0.0027 (4)
N5	0.0344 (7)	0.0215 (6)	0.0219 (6)	−0.0003 (5)	0.0126 (6)	−0.0005 (5)
C1	0.0550 (11)	0.0207 (7)	0.0314 (8)	−0.0003 (7)	0.0247 (8)	0.0026 (6)
C2	0.0387 (9)	0.0165 (7)	0.0310 (8)	−0.0002 (6)	0.0136 (7)	0.0024 (6)
C3	0.0354 (9)	0.0177 (7)	0.0276 (8)	0.0000 (6)	0.0124 (7)	−0.0035 (5)
C4	0.0372 (9)	0.0196 (7)	0.0242 (7)	−0.0003 (6)	0.0143 (7)	−0.0020 (5)
C5	0.0218 (7)	0.0174 (7)	0.0199 (6)	−0.0007 (5)	0.0062 (5)	−0.0001 (5)
C6	0.0251 (7)	0.0165 (7)	0.0192 (7)	−0.0003 (5)	0.0080 (6)	−0.0008 (5)
C7	0.0315 (8)	0.0164 (7)	0.0218 (7)	−0.0015 (5)	0.0107 (6)	−0.0018 (5)
C8	0.0241 (7)	0.0177 (7)	0.0211 (7)	0.0001 (5)	0.0079 (6)	0.0000 (5)
C9	0.0432 (10)	0.0188 (7)	0.0275 (8)	−0.0003 (6)	0.0177 (7)	0.0015 (5)
C10	0.0229 (7)	0.0188 (7)	0.0190 (7)	−0.0008 (5)	0.0066 (6)	−0.0008 (5)
C11	0.0357 (9)	0.0201 (7)	0.0236 (7)	0.0008 (6)	0.0138 (7)	0.0002 (5)
C12	0.0365 (9)	0.0197 (7)	0.0297 (8)	0.0017 (6)	0.0117 (7)	−0.0011 (6)
C13	0.0289 (8)	0.0237 (7)	0.0257 (7)	0.0024 (6)	0.0090 (6)	−0.0063 (6)
C14	0.0360 (9)	0.0277 (8)	0.0223 (7)	0.0000 (6)	0.0141 (7)	−0.0028 (6)

### Geometric parameters (Å, º)

**Table:**

O1—N2	1.3691 (14)	C4—C5	1.3981 (19)
O1—H1	0.93 (2)	C4—H4	0.9500
O2—C7	1.2269 (17)	C5—C6	1.4950 (18)
N1—C1	1.3365 (19)	C6—C7	1.5392 (18)
N1—C5	1.3435 (18)	C8—C10	1.4911 (18)
N2—C6	1.2866 (17)	C8—C9	1.5051 (19)
N3—C7	1.3471 (18)	C9—H9A	0.9800
N3—N4	1.3776 (16)	C9—H9B	0.9800
N3—H3	0.89 (2)	C9—H9C	0.9800
N4—C8	1.2858 (18)	C10—C11	1.3954 (19)
N5—C10	1.3398 (18)	C11—C12	1.3837 (19)
N5—C14	1.3407 (18)	C11—H11	0.9500
C1—C2	1.3831 (18)	C12—C13	1.382 (2)
C1—H1A	0.9500	C12—H12	0.9500
C2—C3	1.3719	C13—C14	1.378 (2)
C2—H2	0.9500	C13—H13	0.9500
C3—C4	1.3861 (17)	C14—H14	0.9500
C3—H3A	0.9500		
			
N2—O1—H1	104.1 (14)	O2—C7—C6	120.28 (12)
C1—N1—C5	119.39 (12)	N3—C7—C6	115.45 (11)
C6—N2—O1	118.04 (11)	N4—C8—C10	115.40 (12)
C7—N3—N4	119.85 (12)	N4—C8—C9	125.33 (12)
C7—N3—H3	115.7 (13)	C10—C8—C9	119.27 (12)
N4—N3—H3	124.4 (13)	C8—C9—H9A	109.5
C8—N4—N3	115.41 (12)	C8—C9—H9B	109.5
C10—N5—C14	117.37 (12)	H9A—C9—H9B	109.5
N1—C1—C2	123.35 (13)	C8—C9—H9C	109.5
N1—C1—H1A	118.3	H9A—C9—H9C	109.5
C2—C1—H1A	118.3	H9B—C9—H9C	109.5
C3—C2—C1	117.44 (8)	N5—C10—C11	122.20 (12)
C3—C2—H2	121.3	N5—C10—C8	116.62 (12)
C1—C2—H2	121.3	C11—C10—C8	121.17 (12)
C2—C3—C4	120.34 (7)	C12—C11—C10	119.04 (13)
C2—C3—H3A	119.8	C12—C11—H11	120.5
C4—C3—H3A	119.8	C10—C11—H11	120.5
C3—C4—C5	118.95 (12)	C13—C12—C11	119.20 (14)
C3—C4—H4	120.5	C13—C12—H12	120.4
C5—C4—H4	120.5	C11—C12—H12	120.4
N1—C5—C4	120.51 (12)	C14—C13—C12	117.83 (13)
N1—C5—C6	116.12 (12)	C14—C13—H13	121.1
C4—C5—C6	123.34 (12)	C12—C13—H13	121.1
N2—C6—C5	128.74 (12)	N5—C14—C13	124.36 (14)
N2—C6—C7	106.58 (11)	N5—C14—H14	117.8
C5—C6—C7	124.62 (11)	C13—C14—H14	117.8
O2—C7—N3	124.27 (13)		

### Hydrogen-bond geometry (Å, º)

**Table:**

*D*—H···*A*	*D*—H	H···*A*	*D*···*A*	*D*—H···*A*
N3—H3···N1	0.89 (2)	1.84 (2)	2.6126 (17)	144 (2)
O1—H1···O2^i^	0.93 (2)	1.98 (2)	2.8327 (14)	151 (2)
O1—H1···N2^i^	0.93 (2)	2.13 (2)	2.8489 (16)	133 (2)
C2—H2···O2^ii^	0.95	2.54	3.1985 (15)	127
C3—H3*A*···O1^iii^	0.95	2.56	3.4755 (15)	163
C13—H13···N5^iv^	0.95	2.46	3.3811 (19)	163

Symmetry codes: (i) −*x*+1, −*y*+2, −*z*; (ii) *x*, *y*−1, *z*; (iii) −*x*+1, −*y*+1, −*z*; (iv) −*x*+1/2, *y*+1/2, −*z*+3/2.
